# Gender and race disparities in weight gain among offenders prescribed antidepressant and antipsychotic medications

**DOI:** 10.1186/s40352-016-0037-7

**Published:** 2016-05-23

**Authors:** Madison L. Gates, Thad Wilkins, Elizabeth Ferguson, Veronica Walker, Robert K. Bradford, Wonsuk Yoo

**Affiliations:** 1 Department of Family Medicine, Medical College of Georgia, Institute of Public and Preventive Health, Augusta University, 1120 15th Street, CJ – 2300, Augusta, GA 30912 USA; 2 Department of Family Medicine, Medical College of Georgia, Augusta University, 1120 15th Street, HB – 4000, Augusta, GA 30912 USA; 3 Department of Psychiatry and Health Behavior, Medical College of Georgia, Augusta University, 997 St. Sebastian Way, Augusta, GA 30912 USA; 4 Lexington Public Library, 3628 Walden Drive, Lexington, KY 40517 USA; 5 Centurion, LLC, 53 Century Blvd, Suite 150, Nashville, TN 37214 USA; 6 Dental College of Georgia, Institute of Public and Preventive Health, Augusta University, 1120 15th Street, CJ – 2300, Augusta, GA 30912 USA

**Keywords:** Antidepressive, Antipsychotic, Body mass index, Mental health, Obesity, Prisoners

## Abstract

**Background:**

Studies have found that antipsychotics and antidepressants are associated with weight gain and obesity, particularly among women and some minority groups. Incarcerated populations (also referred to as offenders, prisoners or inmates) have a high prevalence of mental health problems and 15 % of offenders have been prescribed medications. Despite rates of antidepressant and antipsychotic use, investigations of weight gain and obesity in regard to these agents seldom have included offenders.

**Methods:**

This retrospective descriptive study (2005–2011) was conducted with a Department of Corrections in the east south central United States to investigate the relationship between antidepressant and antipsychotic agents, weight gain, obesity and race or gender differences. We sampled adult offenders who had an active record, at least two weight observations and height data. Offenders were classified into one of four mutually exclusive groups depending upon the type of medication they were prescribed: antidepressants, antipsychotics, other medications or no pharmacotherapy.

**Results:**

The sample population for this study was 2728, which was 25.2 % of the total population. The population not on pharmacotherapy had the lowest baseline obesity rate (31.7 %) compared to offenders prescribed antipsychotics (43.6 %), antidepressants (43.6 %) or other medications (45.1 %). Offenders who were prescribed antidepressants or antipsychotics gained weight that was significantly different from zero, *p* < .001 and *p* = .019, respectively. Women in the antidepressant group gained 6.4 kg compared to 2.0 kg for men, which was significant (*p* = .007). Although women in the antipsychotic group gained 8.8 kg compared to 1.6 kg for men, the finding was not significant (*p* = .122). Surprisingly, there were no significant differences in weight gain between African Americans and Whites in regard to antidepressants (*p* = .336) or antipsychotic agents (*p* = .335).

**Conclusion:**

This study found that women and men offenders prescribed antidepressant or antipsychotic agents gained weight during their incarceration. Women prescribed antidepressants gained significantly more weight than men. However, there was no significant difference in weight gain between African Americans and Whites. Results suggest further investigation is needed to understand the effect of medication history, metabolic syndrome and to explain gender disparities.

## Background

Obesity risk factors are complex and multifaceted, comprised of behaviors related to physical inactivity and excess caloric intake, as well as the environment where people live and social conditions, such as education and poverty (CDC 2015). Along with behavioral, environmental and social factors, studies have found that antipsychotic agents are associated with weight gain and obesity, which is known to increase risk for diabetes, cardiovascular and other chronic diseases (Bai et al. 2015; Ghanizadeh et al. 2013; Henderson et al. 2015; Jafari et al. 2012; Zuo et al. 2015). Antipsychotics also have been linked to the expression of genes related to obesity (Fonseka et al. 2015; Shams & Muller 2014; Tek et al. 2013; Tiwari et al. 2015). Obesity and weight gain also have been associated with the use of antidepressants (Grundy et al. 2014; Heiskanen et al. 2013).

The prevalence of obesity and weight gain are not distributed proportionately across populations, especially in regard to gender and race (Papanastasiou 2013). Antidepressants studies, particularly ones investigating serotonin re-uptake inhibitors, found that women who were prescribed these agents gained more weight and were more likely to be obese or to experience metabolic dysregulation compared to men (Bet et al. 2013; Grundy et al. 2014; Keers & Aitchison 2010; Noordam et al. 2015). Further, women who were prescribed antipsychotics experienced greater weight gain and had more significant metabolic abnormalities than men (Seeman 2010; Softic et al. 2015; Wysokinski et al. 2012). In regard to race, African American patients diagnosed with schizophrenia and prescribed antipsychotic agents gained significantly greater weight compared to White peers (Chan et al. 2013; Krakowski et al. 2009; Stauffer et al. 2010). Contrarily, a few studies have not found a relationship between weight gain and gender or race (Fava et al. 2009). However, studies that have found gender and race disparities related to antidepressant and antipsychotic agents have not been definitive in regard to the mechanisms (genetic expressions and environment) and their relative effects mediating weight gain and obesity (Chan et al. 2013; Keers & Aitchison 2010; Seeman 2010).

Offenders (also referred to as prisoners or inmates in some jurisdictions and countries) also are at risk for disproportionate rates of obesity (Maruschak et al. 2015), along with high prevalence of mental health problems that are treated by medications (Glaze & James 2006). Studies have found that adult offenders, a vulnerable and priority population for poor health, gain weight and become obese during their incarceration, contributing to their increased risk for related conditions, such as diabetes and cardiovascular diseases (Bai et al. 2015; Clarke & Waring 2012; Herbert et al. 2012; Wolff et al. 2012).

Incarcerated populations also have higher prevalence of mental health problems than the general population (Glaze & James 2006; Hassan et al. 2014; Visher & Bakken 2014). The United States Bureau of Justice Statistics’ assessment of mental health in corrections (i.e., prisons) found that 24 % of all offenders incarcerated in state prisons (i.e., lengths of incarceration typically exceeding one year and involving more serious offenses) reported that they had a recent history of a mental health problem (Glaze & James 2006). Large percentages of offenders in state correctional facilities reported having depression (23.5 %) and psychotic symptoms (15.4 %) (Glaze & James 2006). Among offenders in state prisons who had mental health problems, 15 % were prescribed medication (Glaze & James 2006).

The association between weight gain and obesity with antipsychotics and antidepressants primarily has been investigated in non-incarcerated populations, despite the rates in which these medications have been prescribed in corrections. Studies that have included non-incarcerated populations have investigated antipsychotic induced weight gain (AIWG) (Bak et al. 2014; Curtis et al. 2015; Henderson et al. 2015; Shams & Muller 2014; Tek et al. 2013; Tek et al. 2015; Wang et al. 2014), interventions to minimize weight gain for patients taking these agents (Curtis et al. 2015; Ghanizadeh et al. 2013; Jacobowitz et al. 2014; Mahmood et al. 2013; Mizuno et al. 2014; Wu et al. 2012; Zheng et al. 2015) and health disparities in regard to gender, race and ethnicity (Berkowitz & Fabricatore 2011; Chan et al. 2013; Hassan et al. 2014). These studies found a strong relationship between weight gain and use of antipsychotics (Bak et al. 2014; Curtis et al. 2015; Henderson et al. 2015; Shams & Muller 2014; Tek et al. 2013; Tek et al. 2015; Wang et al. 2014) and antidepressants (Berkowitz & Fabricatore 2011; Grundy et al. 2014).

This descriptive study investigated offenders in a state department of corrections to explain the relationship among changes in weight, psychotropic agents (i.e., antidepressants and antipsychotics) and incarceration. The primary goal of this study was to investigate the effect that some psychotropic agents have on changes in weight. We hypothesized the following:

1. Patients who have ever been prescribed either antidepressants or antipsychotics (first or second generation) gain more weight during their incarceration compared to the population that has no history of being prescribed these type of agents (i.e., offenders who are prescribed other medications or no pharmacotherapy).
2. There are gender and race differences in regard to weight gain for patients who have ever been prescribed either antidepressants or antipsychotics.
  - Women who have ever been prescribed antidepressants or antipsychotics gain more weight than men.
  - African Americans who have ever been prescribed antidepressants or antipsychotics gain more weight than Whites.

## Methods

This retrospective descriptive study was approved by an institutional review board (IRB), Protocol number: 10-0382F2L, at an academic health center and was conducted for 2005–2011 in collaboration with a Department of Corrections (DOC) in the east south central region of the United States.

The DOC for this study had an electronic health record (EHR), from which all health data (diagnoses, medication classification, height and weight values) were extracted. Clinic staff created an electronic record for all offenders upon their arrival at the DOC reception center, an assessment facility where everyone received a complete physical, mental health and dental examination. The DOC also used an offender management system (OMS), an electronic tracking and case management system, which collected demographic, social, criminal record (offense), sentence and parole data. The offender management record was created electronically and managed by correctional officers and case managers.

### Inclusion and exclusion criteria

The data extraction criterion for the EHR and OMS was active records (i.e., offenders currently incarcerated) between June 1, 2005 and December 31, 2010. The date range was determined by the implementation of the DOC’s EHR, which occurred in 2005. All adult offenders (men and women) were eligible for inclusion. Offenders were excluded if they did not have at least two weight observations, a baseline and post baseline value (last recorded weight), and if they did not belong to a mutually exclusive medication group. Offenders also were excluded if there were no valid height data and their length of incarceration was not greater than zero. Offenders who met the inclusion criteria were assigned to a medication group based on pharmacy data identifying whether or not an offender had been prescribed an antidepressant or antipsychotic. These data allowed us to create four mutually exclusive groups: antidepressants, antipsychotics, other medications or no pharmacotherapy.

### Data collection

Health record data for this study were primarily structured (i.e., users selected or entered data into the record, using standardized data dictionaries). Prescriptions were generated electronically using the National Drug Code (NDC). Diagnoses were entered using International Statistical Classification of Diseases (ICD-9) and supplemented by the Systematized Nomenclature of Medicine (SNOMED). The structured nature of prescriptions and diagnoses provided a degree of standardization. Clinic staff, primarily nurses, entered height and weight data. Data from the OMS were primarily entered by correctional officers into discreet fields. Race, ethnicity and gender were pre-populated based on court records.

Anthropometric data, such as body mass index (BMI), were not available in the extracted data; BMI was calculated as weight in kilograms (kg) divided by height in meters squared (BMI = weight in kg/height in m^2^). The calculated BMIs were classified using the World Health Organization’s (WHO) cut-off points (WHO 2004), as shown in Table 1. Percent weight change [(last weight – baseline-weight)/baseline weight] was calculated and used to determine whether or not offenders gained medically significant weight (weight gain ≥ 7 % of baseline weight) (Arterburn et al. 2015; Bak et al. 2014; Curtis et al. 2015; Lencz & Malhotra 2009; Maayan & Correll 2010; Stauffer et al. 2010; Tek et al. 2015).

**Table 1:** WHO BMI Classification (WHO 2004)

Classification	Cut-off point
Underweight	<18.5
Normal weight	18.5–24.9
Overweight	25.0–29.9
Obesity Class I	30.0–34.9
Obesity Class II	35.0–39.9
Obesity Class III	≥40.0

### Statistical analyses

SAS® 9.4 (SAS Institute, Cary, NC) was used to conduct statistical tests. Frequencies and percentages were conducted for nominal and categorical variables, such as race, gender, BMI classification, and medications. Means and standard deviations (SD) were calculated for continuous variables, including BMI, and percent weight change, length of time between weight observations. Chi-square (χ^2^) was computed to evaluate whether or not populations have different proportions and odds ratios were computed to determine if the four mutually exclusive medication groups (antidepressant, antipsychotic, other medication and no pharmacotherapy) differed in regard to exposure (medication use) and response (changes in weight). Differences in baseline and last weight observation (end of study) were examined using paired *t*-test to determine if the different groups on average gained weight that was statistically significant from zero. The paired *t*-test was performed for each group. Group comparisons for race and gender were made using the Wilcoxon rank-sum test. Comparisons for race were limited to African Americans and Whites, since there were too few other race and ethnic populations for meaningful analyses. The Kruskal-Wallis test was computed to evaluate differences among the four medication groups. To adjust for multiple comparisons and the increased probability of a Type I error (a false positive), we performed the Bonferroni correction, which evaluates the significance of pairwise comparisons using an adjusted alpha (α/n, where *n* = number of pairwise comparisons).

## Results

This study sampled 2728 offenders (25.2 %) out of a population of 10,841 who met the inclusion criteria (e.g., valid weight and height observations). A large percent of the population was excluded for the following reasons: 1) medication groups were mutually exclusive; 2) offenders without chronic diseases did not report to clinics regularly and did not always have multiple weight observations; 3) patients primarily seen for mental health issues did not have vital statistics recorded in the health record on a consistent basis; 4) a segment of the population had not been incarcerated long enough to have multiple weight values.

Despite excluding a large percent of the population, African Americans and offenders prescribed antidepressants or antipsychotics were overrepresented in the study sample; however, the percent of women offenders was less than the total population (Table 2). Although the study sample and total population differed statistically (e.g., race and gender), both groups were majority White, male, not prescribed any medication and had a primary offense related to violence (Table 2). The mean age was 40.2 for the study sample and 37.5 for the total population (Table 2).

**Table 2:** Differences between Study and Total Population

Variable	Study	Total	*p*
	*n* (%) or Mean ± SD	*n* (%) or Mean ± SD	
Race			
African American	897 (34.2)	3,192 (29.4)	< .001
Asian	2 (0.1)	11 (0.1)	.423
Hispanic	11 (0.4)	150 (1.4)	< .001
American Indian	2 (0.1)	12 (0.1)	.625
White	1,657 (63.2)	7,175 (66.2)	.004
Unknown	53 (2.0)	297 (2.7)	.039
Gender			
Men	2,458 (93.8)	9,767 (90.1)	< .001
Women	164 (6.3)	1074 (9.9)	< .001
Age	40.20 ± 10.3	37.5 ± 11.5	< .001
Violent Offense	864 (48.2)	3,305 (48.4)	.901
Medications			
Psychotropic	390 (14.9)	1,446 (13.3)	.040
Other medications	823 (31.4)	4,487 (41.4)	< .001
No pharmacotherapy	1,409 (53.7)	6,136 (56.6)	.007

Table 3 shows that medication groups had similar BMIs at baseline and last observation; however, the no pharmacotherapy group had the lowest BMI (28.0) compared to a BMI ≥ 30 for the other groups. A comparison of the three medication groups (antidepressants, antipsychotics and other medications) indicated that baseline BMI was not significantly different (*p =* .353). The three groups also did not have significantly different last observed BMI (*p* = .913). Further, when antipsychotics (first generation and second generation) were compared, their baseline BMI was not significantly different (*p* = .860); last observed BMI also was not significantly different (*p* = .795) between the two subgroups. Table 3 also shows that the average length of time between baseline and last observed BMI for the three groups prescribed a medication ranged from 2.3 to 2.5 years, which was not significantly different (*p* = .204). When the length of time between baseline and last observed BMI for first generation antipsychotics (FGA) and second generation antipsychotics (SGA) were compared (Table 3), the two subgroups also were not significantly different (*p* = .143).

**Table 3:** BMI by Medication Group: Baseline and Last Observation

		Baseline BMI		Last observation		Duration^a^	
			95 % CI^b^		95 % CI		95 % CI
Medication Group	Number	Mean (SD)	[Lower, Upper]	Mean (SD)	[Lower, Upper]	Mean (SD)	[Lower, Upper]
Antidepressants	324	30.0 (5.4)	[29.4, 30.6]	30.9 (5.4)	[30.3, 31.5]	2.3 (1.1)	[2.2, 2.4]
Antipsychotics	172	30.0 (6.0)	[29.1, 30.9]	30.8 (5.2)	[30.0, 31.5]	2.4 (1.1)	[2.6, 2.6]
Other medication	823	30.4 (5.6)	[30.0, 30.8]	30.7 (5.1)	[30.3, 31.0]	2.5 (1.1)	[2.4, 2.5]
No pharmacotherapy	1409	28.0 (4.6)	[28.6, 29.1]	29.3 (4.4)	[29.1, 29.5]	1.9 (1.1)	[1.9, 2.0]
Antipsychotics							
FGA	99	29.9 (5.7)	[28.8, 31.1]	30.5 (4.9)	[29.6, 31.5]	2.3 (1.1)	[2.1, 2.63]
SGA	73	30.0 (6.3)	[28.5, 31.5]	31.0 (5.6)	[29.7, 32.4]	2.6 (1.0)	[2.4, 2.8]

^a^ Length of time (years) between baseline and last weight observation

^b^95 % Confidence Interval

There were few offenders entering corrections who had a BMI classified as normal or underweight, as shown in Table 4. A plurality of offenders across medication groups (antidepressant, antipsychotic and other medications) entered corrections obese. As Table 4 shows, the population not prescribed pharmacotherapies entered corrections with the lowest obesity rate (31.7 %) compared to offenders who used antipsychotics (43.6 %), antidepressants (43.6 %) or other medications (45.1 %). Patients on antidepressants had the greatest prevalence for Obesity Class III at baseline.

**Table 4:** Distribution of World Health Organization’s BMI Classification on Entry into Corrections

	Antidepressants		Antipsychotics		Other medications		No pharmacotherapy	
Classification	Baseline	Last observation	Baseline	Last observation	Baseline	Last observation	Baseline	Last observation
	*n* (%)	*n* (%)	*n* (%)	*n* (%)	*n* (%)	*n* (%)	*n* (%)	*n* (%)
Underweight	0 (0)	0 (0)	0 (0)	0 (0)	2 (0.2)	1 (0.1)	2 (0.1)	0 (0)
Normal	46 (14.2)	24 (7.4)	32 (18.6)	12 (7)	89 (10.8)	55 (6.7)	247 (17.5)	189 (13.4)
Overweight	137 (42.3)	154 (47.5)	65 (37.8)	84 (48.8)	361 (43.9)	385 (46.8)	712 (50.5)	733 (52)
Obesity I	98 (30.2)	87 (26.9)	49 (28.5)	44 (25.6)	236 (28.7)	254 (30.9)	316 (22.4)	342 (24.3)
Obesity II	21 (6.5)	38 (11.7)	15 (8.7)	21 (12.2)	84 (10.2)	88 (10.7)	99 (7)	104 (7.4)
Obesity III	22 (6.8)	21 (6.5)	11 (6.4)	11 (6.4)	51 (6.2)	40 (4.9)	33 (2.3)	41 (2.9)
	324		172		823		1409	

As Table 5 shows, the list of prescribed antidepressants and antipsychotics was not extensive. Amitriptyline was the most frequently prescribed antidepressant. Risperidone and perphenazine were the most often prescribed SGA and FGA, respectively, while olanzapine was seldom used and no offender in the study used clozapine (See Table 5). Further, FGA were prescribed more often than SGA.

**Table 5:** Antidepressant and Antipsychotic Medications Prescribed to Offenders during 2005–2010

Medication class	Generic name	Number	% of all psychotropics	% of sample
Antidepressant	Amitriptyline	95	19.2	3.6
	BuPROPion	34	6.9	1.3
	Citalopram	53	10.7	2
	Duloxetine	1	0.2	0
	Fluoxetine	45	9.1	1.7
	Mirtazapine	52	10.5	2
	Paroxetine	19	3.8	0.7
	Sertraline	13	2.6	0.5
	Venlafaxine	12	2.4	0.5
	Total	324	65.3	12.4
Antipsychotic				
FGA	ChlorproMAZINE	25	5.0	1
	Fluphenazine	5	1.0	0.2
	Haloperidol	20	4.0	0.8
	Loxapine	2	0.4	0.1
	Perphenazine	42	8.5	1.6
	Thiothixene	5	1.0	0.2
	Total	99	20.0	3.8
SGA	Aripiprazole	4	0.8	0.2
	Olanzapine	3	0.6	0.1
	Quetiapine	8	1.6	0.3
	Risperidone	55	11.1	2.1
	Ziprasidone	3	0.6	0.1
	Total	73	14.7	2.8

The paired *t*-test was performed for all medications groups to indicate whether or not offenders in their respective group had changes in weight that was significantly different from zero. The test indicated that last observed weight was greater than baseline for the population prescribed antidepressants (*p* < .001), antipsychotics (*p* = .019), other medications (*p* = .018) and the no pharmacotherapy group (*p* < .001). In regard to the antipsychotic subgroups, changes in baseline and last observed weight was only significant for the population prescribed SGA (*p* = .033); FGA had a *p*-value = .209.

Women and men, who were taking antidepressants, had weight gain significantly different from zero *p* < .001 and *p* = .001, respectively, as did African Americans and Whites, *p* = .030 and *p* < .001, respectively. Although women and men gained weight while taking antipsychotics, changes between baseline and last weight observed were not significant (*p* = .066 and *p* = .093, respectively). African Americans and Whites, who were prescribed antipsychotics, also gained weight, but the increase was not significantly different from zero (*p* = .148 and *p* = .081, respectively).

The four medication groups also had significantly different proportions (*p* < .001) in regard to medically significant weight gain. Larger proportions of patients taking antidepressants or antipsychotics (32.7 and 33.1 %, respectively) gained medically significant weight compared to those on other medications or no pharmacotherapy (22.6 % for both groups). There was no significant difference in proportions (*p* = .212) for medically significant weight gain between patients prescribed FGA or SGA.

Table 6 shows that offenders prescribed antidepressants gained more weight compared to the antipsychotic, other medications and no pharmacotherapy groups. Further, the Kruskal-Wallis test indicated that weight change, percent weight change and change in BMI were significantly different (*p* = .027, *p* = .027 and *p* = .021 respectively) across the four medication groups. Weight change, percent weight change and change in BMI are highly collinear, given that all the variables are derived from weight observations and evidenced by the similarity in *p*-values; thus, subsequent analyses focus on weight change.

**Table 6:** Changes in Weight and BMI for Patients Using Antidepressants and Antipsychotics

		Weight change (kg)		Percent weight change		Change in BMI	
			95 % CI§		95 % CI		95 % CI
Medication group	Number	Mean (SD)	[Lower, Upper]	Mean (SD)	[Lower, Upper]	Mean	[Lower, Upper]
Antidepressants	324	2.7 (10.1)	[1.6, 3.8]	3.8 (11.8)	[2.6, 5.1]	0.9 (3.4)	[0.6, 1.3]
Antipsychotics	172	2.3 (12.6)	[0.4, 4.2]	4.2 (16.1)	[1.8, 6.7]	0.8 (4.3)	[0.1, 1.4]
Other medications	823	0.8 (9.6)	[0.1, 1.5]	1.6 (10.2)	[0.9, 2.3]	0.3 (3.1)	[0.1, 0.5]
No pharmacotherapy	1409	1.4 (8.1)	[1, 1.8]	2.1 (9.5)	[1.6, 2.6]	0.4 (2.5)	[0.3, 0.6]
Antipsychotics							
FGA	99	1.6 (12.8)	[−0.9, 4.2]	3.6 (16)	[0.4, 6.8]	0.6 (4.3)	[−0.3, 1.4]
SGA	73	3.1 (12.3)	[0.3, 6]	5.1 (16.2)	[1.4, 8.9]	1 (4.2)	[0.1, 2]

§ 95 % Confidence Interval

**Fig. 1:**
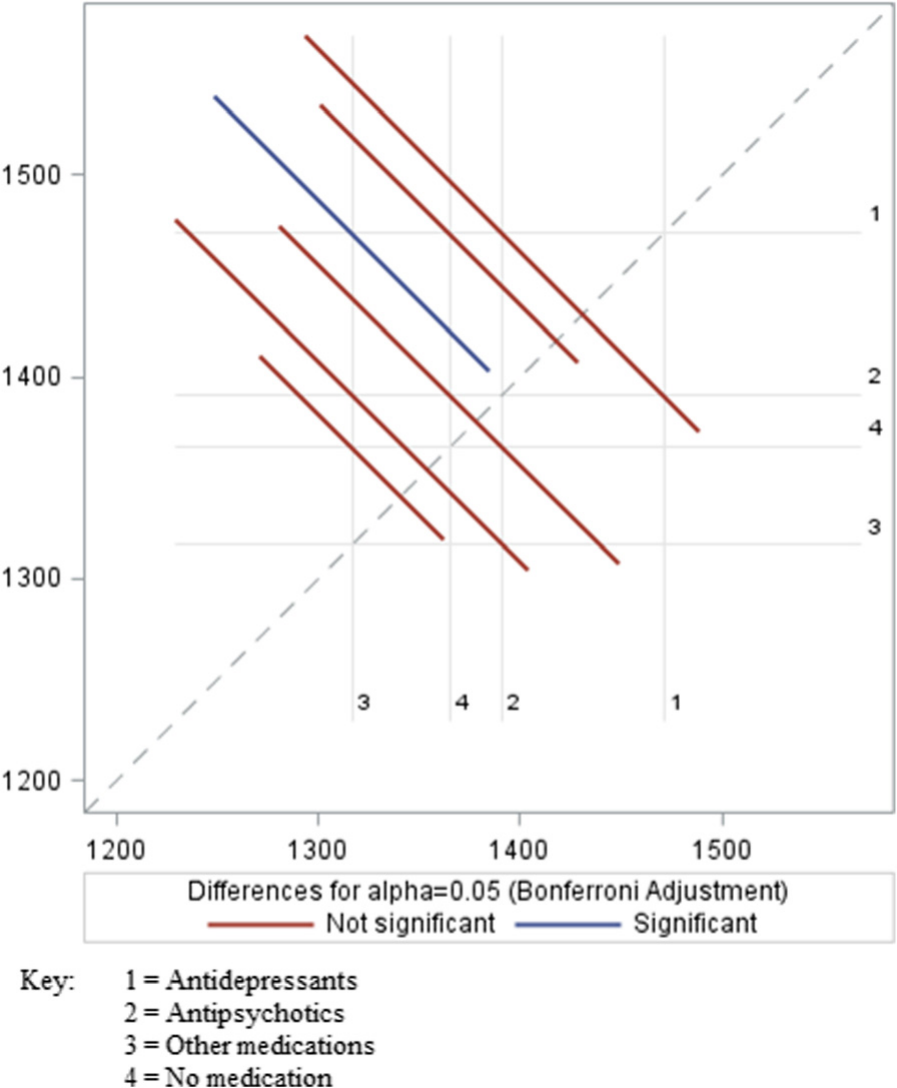
Bonferroni Adjustment – Significant and Not Significant Comparisons

Accounting for multiple comparisons (i.e., four medication groups), we performed a Bonferroni correction to identify which groups had significantly different weight changes from one another (Table 7). The test indicated that offenders in the antidepressants group had significantly different weight changes compared to patients prescribed other medications. Although the antidepressants – other medications comparison was the only one that satisfied the adjusted significance level, Fig. 1 shows that antidepressants compared to no pharmacotherapy was near the cut-off point for significance.

**Table 7:** Bonferroni Adjustment for Multiple Comparisons

Least square means		
Comparisons	Differences in means	95 % confidence limits [lower, upper]
Antidepressants - Antipsychotics	80.1	[−115.7, 275.9]
Antidepressants - Other medications^a^	154.5	[18.4, 290.6]
Antidepressants - No medication	106.2	[−21.6, 234.1]
Antipsychotics - Other medications	74.4	[−99.6, 248.4]
Antipsychotics - No medication	26.1	[−141.5, 193.7]
Other medications - No medication	−48.3	[−139.3, 42.8]

^a^Significant

When the antipsychotic subgroup was examined, offenders prescribed SGA gained 1.5 kg more weight compared to the FGA group (Table 6), however this finding was not significant (*p* = .448).

African Americans, who were prescribed antidepressants, on average, gained the same weight (2.6 kg) as Whites taking the same class of medication, which was not significant (*p* = .534). In regard to antipsychotics, African Americans compared to Whites gained more weight (3.0 to 2.0 kg, respectively), but this differences was not significant (*p* = .157). There were no significant weight change differences between African Americans and Whites when the antipsychotic subgroup was examined. African Americans in the FGA group gained 2.1 kg compared to 1.3 kg for Whites (*p* = .313). In regard to SGA, African Americans also gained more weight than Whites (4.0 and 2.8 kg, respectively), *p* = .355. While there were a few significant differences between African Americans and Whites in regard to length of time between baseline and last observed weight (i.e., antidepressant, *p* = .035, and FGA, *p* = .034), the difference for the antidepressant and FGA groups were 4 and 6 months, respectively. There were no race differences for length of time between baseline and last observed weight for other medication groups.

The Wilcoxon two-sample test indicated that gender was significant (See Table 8). Women offenders who were prescribed antidepressants had greater increases in weight, percent weight change and BMI compared to men, as shown in Table 8. However, there were no significant differences for weight change in regard to gender and antipsychotics. Although women compared to men gained much more weight, there were no significant differences between FGA and SGA use (Table 8). In regard to length of time between baseline and last recorded weight, there were few significant differences between women and men (Table 8). Although there were significant gender differences in regard to duration between baseline and last observed weight for the antipsychotic group (*p* = .027), the difference was 6 months (Table 8).

**Table 8:** Antidepressants, Antipsychotics and Gender Differences

	Women		Men		
		95 % confidence interval		95 % confidence interval	
	Mean (SD)	[Lower, Upper]	Mean (SD)	[Lower, Upper]	*p*
Antidepressant	*n* = 53		*n* = 271		
Weight change (kg)	6.4 (11.2)	[3.5, 10.0]	2.0 (9.7)	[0.8, 3.3]	0.007
Percent weight change	8.5 (15.7)	[4.5, 13.6]	2.9 (10.6)	[1.7, 4.4]	0.012
BMI change	2.5 (4.4)	[1.3, 3.9]	0.6 (3.0)	[0.3, 1.1]	0.004
Duration (years)†	2.1 (0.9)	[1.8, 2.3]	2.4 (1.1)	[2.2, 2.5]	0.055
Antipsychotic	*n* = 16		*n* = 156		
Weight change (kg)	8.8 (17.8)	[−0.6, 18.3]	1.6 (11.8)	[−0.3, 3.7]	0.122
Percent weight change	13.9 (29.6)	[−1.9, 29.6]	3.3 (13.8)	[1.2, 5.8]	0.166
BMI change	3.4 (7.2)	[−0.4, 7.3]	0.5 (3.8)	[−0.1, 1.1]	0.126
Duration (years)	1.9 (1.0)	[1.3, 2.4]	2.5 (1.0)	[2.3, 2.7]	0.027
FGA	*n* = 7		*n* = 92		
Weight change (kg)	10.8 (17.2)	[−5.1, 26.6]	0.9 (12.2)	[−1.7, 3.7]	0.096
Percent weight change	15.8 (31.4)	[−13.3, 44.8]	2.7 (14.1)	[−0.3, 6.0]	0.142
BMI change	4.1 (7.2)	[−2.5, 10.8]	0.3 (3.9)	[−0.5, 1.2]	0.094
Duration (years)	1.7 (1.0)	[0.7, 2.6]	2.4 (1.1)	[2.2, 2.6]	0.093
SGA	*n* = 9		*n* = 64		
Weight change (kg)	7.3 (19.2)	[−7.4, 22.1]	2.5 (11.1)	[−0.2, 5.6]	0.568
Percent weight change	12.4 (29.8)	[−10.5, 35.3]	4.1 (13.3)	[0.8, 7.8]	0.633
BMI change	2.9 (7.6)	[−3.0, 8.8]	0.8 (3.5)	[−0.1, 1.7]	0.609
Duration (years)	2 (1.1)	[1.2, 2.8]	2.7 (1.0)	[2.4, 2.9]	0.100

**†** Length of time between baseline and last observed weight

Within the population of women offenders, there were no significant differences among women who were taking antidepressants (6.4 kg), antipsychotics (8.8 kg), other medications (4.3 kg) or no pharmacotherapy (3.7 kg) in regard to changes in weight (*p* = .423), even though women prescribed antipsychotics gained the most weight across groups. Further, women on FGA gained more weight than women taking SGA (10.8 and 7.3 kg, respectively); however, this differences was not significant (*p* = 1.0). There were no significant differences for weight change (*p* = .122) within men who were prescribed antidepressants (2.0 kg), antipsychotics (1.6 kg), other medications (0.6 kg) or no pharmacotherapy (1.3 kg). FGA and SGA differences for changes in weight for men also were not significant (*p* = .444), even though men gained more weight on SGA than men on FGA (2.5 and 0.9 kg, respectively).

## Discussion

This study found that women and men offenders who were prescribed antidepressants or antipsychotics (FGA and SGA) gained weight during their incarceration over similar lengths of time, but the antidepressant group had weight gain that was significantly different from patients taking other medications. Weight gain differences between the antidepressant and no pharmacotherapy groups approached the significance level. Further, there was no significant difference in weight gain between SGA and FGA, even though SGA resulted in greater weight gain than FGA. There were gender differences in regard to antidepressant use where women gained significantly more weight compared to men; however, gender was not significant for antipsychotics (FGA and SGA). Differences in weight changes between African Americans and Whites were not significant for any of the psychotropic agents.

Our results are consistent with previous studies, primarily outside of corrections, which found an association between weight gain and antidepressants (Berkowitz & Fabricatore 2011; Grundy et al. 2014) and antipsychotics (Bak et al. 2014; Jafari et al. 2012; Mahmood et al. 2013; Panariello et al. 2012; Tek et al. 2015; Tiwari et al. 2015; Wang et al. 2013). Studies have found that the relationship between mental health, particularly depression, and weight gain and obesity is greater for women than men (Berkowitz & Fabricatore 2011; Grundy et al. 2014), which is similar to the findings of this study where women gain significantly more weight than men. Race has been found to be an explanatory factor for differences in AIWG, in which African Americans who take these agents have greater rates or odds of gaining weight compared to Whites and other groups (Chan et al. 2013; Krakowski et al. 2009; Stauffer et al. 2010). However, this study did not find a significant difference in weight gain between African American and White offenders. Further, weight gain experienced by women on SGA was less than FGA, which is not consistent with studies attributing greater weight gain with SGA; however, men had greater weight gain with SGA, which was expected (Lencz & Malhotra 2009; Maayan & Correll 2010; Shams & Muller 2014; Zuo et al. 2015).

The finding that antipsychotics, particularly SGA, were not associated with significant or greater weight in comparison to FGA may be related to the absence of clozapine and very few offenders prescribed olanzapine, which have been found to contribute to weight gain more so than other SGA (Arterburn et al. 2015; American Diabetes Association et al. 2004; Jafari et al. 2012; Krakowski et al. 2009; Shams & Muller 2014; Tek et al. 2015; Wang et al. 2013). FGA for this DOC also were prescribed more often than SGA. Further, it has been found that women prescribed risperidone, of which a plurality of women offenders in this DOC used, experienced greater weight gain in comparison to men (Hung et al. 2010), which may explain, in part, the SGA gender disparity. Thus, the SGA gender findings may be explained by the heterogeneity of these drugs in regard to weight gain (Softic et al. 2015; ÜÇOk & Gaebel 2008), but the greater weight gain among women who used FGA compared to SGA requires further investigation.

Antidepressant (Barnard et al. 2013; Kivimäki et al. 2011; Pan et al. 2012; Wu et al. 2014; Yoon et al. 2013) and antipsychotic (Chaggar et al. 2011; American Diabetes Association et al. 2004; Fernandez-Egea et al. 2011; Goff et al. 2005; Green et al. 2015; Ward et al. 2013; Wysokinski et al. 2012) studies have found these agents a risk factor for metabolic syndrome or dysregulation. The risk of type 2 diabetes was associated with antidepressant and antipsychotic use, as these agents disrupted glucose metabolism (Barnard et al. 2013; Fernandez-Egea et al. 2011; Green et al. 2015; Mahmood et al. 2013; Wang et al. 2013; Wysokinski et al. 2012). Studies also found that some antipsychotic agents (e.g., olanzapine) were associated with increases in low-density lipoprotein, triglycerides and total cholesterol (Fernandez-Egea et al. 2011; Wysokinski et al. 2012). Increased risk of type 2 diabetes and dyslipidemia related to antidepressant and antipsychotic use may have a moderating effect on weight gain, particularly for SGA, since offenders who developed these diseases are more likely to have encounters with primary care physicians, increasing the likelihood of weight management.

The EHR for this DOC was used by psychiatrists, primary care physicians and mental health professionals engaged in the care of offenders diagnosed with depression or psychotic symptoms. The EHR provided psychiatrists and primary care physicians full access to the medication history, diagnosis list, vital statistics and lab data for patients they shared. Thus, mental and physical health were not provided in isolation from one another or dependent upon patients to communicate their health history to the two specialties. While the EHR may not have embodied multidisciplinary healthcare teams, it decreased barriers in regard to specialties accessing patients’ complete health history. Further, offenders with depression or psychoses, who also had chronic diseases, were seen minimally 3–4 times per year, depending upon national standards and disease control. Chronic disease clinics provided an opportunity for primary care physicians to intervene or counsel offenders who gain weight.

## Conclusion

Providing mental health treatment in a correctional environment is complex, especially given the focus on safety and security of offenders, correctional staff and the public. However, there are structural advantages for providing healthcare in a closed environment, such as corrections, which has extensive knowledge about its patient population and is able to assign patients to chronic disease clinics. Findings from the literature also posit that mental health and incarceration is complex whereby some offenders perceive the prison environment as a contributor to their poor mental health status whereas others see corrections as an opportunity for healthcare services (Goomany & Dickinson 2015; Walker et al. 2014). While beyond the scope of this study, offenders in this DOC received mental health pharmacotherapy in similar proportions to national data (Glaze & James 2006), but it is unknown why there was greater use of FGA compared to SGA.

SGA have been found to have fewer extrapyramidal side effects, such as Parkinsonian and akathisia, and a lower risk of tardive dyskinesia compared to FGA (Gallego et al. 2012; Lencz & Malhotra 2009; ÜÇOk & Gaebel 2008), but typically are more expensive (Gallego et al. 2012). Conversely, metabolic syndrome and weight gain side effects have been associated most strongly with SGA (Gallego et al. 2012). Further, it has been posited that FGA side effects (e.g., extrapyramidal) are attenuated by low dose, and the comparative effectiveness of SGA in regard to FGA is debated (Gallego et al. 2012). Thus, corrections may be managing its population effectively while also containing cost. Correctional systems understand the importance of providing care to offenders who have mental health diagnoses, even if the perspective is based on security concerns, that is, corrections does not have the luxury of having untreated offenders with depression or psychotic symptoms; this would be counter to maintaining security. However, gender disparities, particularly to the detriment of women, is a recurrent finding in correctional health studies, especially as it relates to weight gain or obesity (Clarke & Waring 2012; Gates & Bradford 2015; Herbert et al. 2012).

The emerging findings of this study suggest promise, but also an opportunity to intervene particularly in the health of women offenders who are prescribed antidepressant and/or antipsychotic agents. Based on the preliminary findings of this study, there are several recommendations that we propose for the short and long-term. The intake process typically includes multiple health screenings and assessment tools. We propose that departments of corrections review its screening tools and procedures related to the use of psychotropic agents to determine if there are weight management interventions, particularly for the population most at risk. Further investigations of antidepressant and antipsychotic related weight gain likely will result in screening tools becoming risk assessment or predictive instruments, which we would recommend corrections adopt. Corrections, in collaboration with academic partners, likely will improve the mental and physical health of its population by learning more about the psychosocial factors of the population prescribed psychotropic agents, which may address underlying mental health issues, including the effects of incarceration.

### Limitations

This study is among the few studies to have investigated psychotropic agents (i.e., antidepressants and antipsychotics) and weight gain in corrections. There were several limitations for this study, principally, the lack of medication history for offenders during their incarceration. Studies investigating antidepressant and antipsychotic use have found dose (ÜÇOk & Gaebel 2008) and duration (Bak et al. 2014; Tek et al. 2015) factors related to weight gain. A more extensive and complete medication and mental health history would have identified which offenders were antidepressant and antipsychotic naïve patients and what effect medication switching, polypharmacy or mental health severity may have had. In addition to antidepressant and antipsychotic medication history, this study did not collect adherence data to evaluate whether or not adherence correlated with weight gain or explained findings inconsistent with the literature, such as greater weight gain from FGA compared to SGA. Other studies have found antidepressant and antipsychotic medications are a contributor to metabolic syndrome, but this study did not investigate the weight gain effect that these drugs may have had in regard to risk for related chronic diseases. The inclusion and exclusion criteria resulted in a large percent of missing data for women offenders (a 3.7 % under sampling for the group when compared to the total population).

This study focused on descriptively investigating the effect of antidepressant and antipsychotic induced weight gain with a focus on race and gender differences. The findings of this study suggest further investigation is needed to understand the effect of mental health severity, medication history, metabolic syndrome and to explain gender disparities. Multivariable analyses will be needed to adjust for the effects from confounding factors with appropriate collinearity procedure (Yoo et al. 2014). Furthermore, data-mining methods like tree-based approaches or random forests may effectively capture moderation effects associated with antidepressant and antipsychotic use and weight gain (Yoo et al. 2012).

## Abbreviations

AIWG: antipsychotic induced weight gain
BMI: body mass index
DOC: Department of Corrections
EHR: electronic health record
FGA: first generation antipsychotic
ICD: international statistical classification of diseases
NDC: National Drug Code
OMS: offender management system
SGA: second generation antipsychotic
SNOMED: systematized nomenclature of medicine
WHO: World Health Organization
